# *Calamagrostis hongii* (Poaceae, Agrostidinae), a new species from southwestern China

**DOI:** 10.3897/phytokeys.161.53010

**Published:** 2020-10-29

**Authors:** Bing Liu, Beata Paszko

**Affiliations:** 1 State Key Laboratory of Systematic and Evolutionary Botany, Institute of Botany, Chinese Academy of Sciences, Beijing 100093, China; 2 Sino-Africa Joint Research Center, Chinese Academy of Sciences, Wuhan 430074, China; 3 W. Szafer Institute of Botany, Polish Academy of Sciences, Lubicz 46, PL-31-512 Kraków, Poland

**Keywords:** Asia, *
Deyeuxia
*, distribution, endemism, Flora of China, Sino-Himalayan region, taxonomy

## Abstract

*Calamagrostis hongii*, a new species of *Calamagrostis* (Poaceae) from southwestern China (S Chongqing, W Guizhou, Sichuan, SE Xizang, Yunnan), is here described and illustrated. It is similar to *C. arundinacea* and *C. effusiflora* in spikelet traits, but can be distinguished by its moderately or densely scabrous upper leaf surface with ribs covered by short, stiff, prickle hairs, and glabrous leaf sheaths, blades and collars. Nomenclature *Deyeuxia zhongdianensis* lacks Latin description or diagnosis and is an unavailable *nomen nudum* (naked name).

## Introduction

Six species of *Calamagrostis* Adans. and thirty four species of *Deyeuxia* Beauv. were reported in the published taxonomic treatments for the “Flora of China” (Lu and Phillips 2006; Lu et al. 2006). Among them, 15 species of *Deyeuxia* and one species of *Calamagrostis* were considered to be endemic to China (Lu and Phillips 2006; Lu et al. 2006; Huang et al. 2011, 2017). Twelve of these endemics occur in the mountains of SW China, which are recognized as a global biodiversity hotspot (Boufford 2014; Cai et al. 2019) and this region continues to produce species of grass new to science. Recent examples include one new species of *Achnatherum* Beauv., two new species of *Deyeuxia*, two new species of *Ptilagrostis* Griseb. and two new species of *Stipa* L. (Paszko and Chen 2013; Paszko and Pendry 2013; Nobis et al. 2016; Zhang et al. 2016, 2017, 2018; Zhao and Guo 2017; Cai et al. 2019).

At present, a taxonomic revision of *Calamagrostis* (including Asian species of *Deyeuxia*) for China is being prepared by the second author (Paszko 2019). Following recent molecular studies, all Asian species of *Deyeuxia* have been placed in the genus *Calamagrostis* (Saarela et al. 2010, 2017; Paszko et al. 2017). To date, a great number of major changes have been made within the classification of the genus *Calamagrostis* in China since 2006. These changes cover new species, taxonomic novelties and range extensions of several species. There is a considerable increment in the number of species. Thus, the total number of species described and reported from China increased from 40 up to 47. The subsequent major changes are summarized in more detail herein. To date, two species (*C. sorengii* (Paszko & WL Chen) Paszko and *C. gaoligongensis* (Paszko) Paszko) new to science have been described (Paszko and Chen 2013; Paszko and Pendry 2013), a third one is described here (*C. hongii* Paszko & Bing Liu). *Calamagrostis altaica* Tzvelev, described from China and overlooked in the “Flora of China”, was shown to be a separate species (Paszko et al. 2016a). Two names, *Calamagrostis kengii* T. F.Wang and *Deyeuxia flavens* Keng, have been considered synonymous with taxa that occur beyond China (Paszko 2012; Paszko and Ma 2011).

In addition, several major range extensions to China and beyond turn up. Two new records for China (*C. filiformis* Hooker f., *C. garhwalensis* Hubbard & Bor) have been reported (Paszko 2012, 2014b) and Tzvelev’s (1968) records of *C. salina* Tzvelev, neglected in the “Flora of China”, have been confirmed from China (Paszko et al. 2016b). Three species, *Calamagrostis effusiflora* (Rendle) J.L. Yang, *C. diffusa* (Keng) Keng f. and *C. himalaica* (Liou ex W.L. Chen, emend. Paszko) Paszko, can no longer be recognized as endemic to China because they have been documented in at least one additional country (Paszko and Soreng 2013; Paszko 2014a, Paszko in Nobis et al. 2015).

*Calamagrostis* has its highest species diversity in SW China, particularly in Sichuan, SE Xizang and Yunnan, where the species number of *Calamagrostis* has increased recently. Several species have much wider distribution in China than previously thought. To date, 14 new provincial records have been reported from this region including seven from Yunnan (*C. debilis* Hook. f., *C. extremiorientalis* (Tzvelev) Prob., *C. filiformis* Griseb., *C. gaoligongensis* (Paszko) Paszko, *C. himalaica* (Liou ex W.L.Chen emend. Paszko) Paszko, *C. nyingchiensis* (Kuo & Lu) Paszko, *C. yanyuanensis* Yang), four from Xizang (*C. abnormis* (Hook. f.) Shukla, *C. gaoligongensis* (Paszko) Paszko, *C. sichuanensis* Yang, *C. sorengii* (Paszko & W.L.Chen) Paszko) and three from Sichuan (*C. abnormis*, *C. garhwalensis* C.E.Hubb. & Bor, *C. extremiorientalis* (Tzvelev) Prob.) (Paszko 2012, 2014a, 2015, 2016, 2019; Paszko and Chen 2013; Paszko and Pendry 2013; Paszko and Soreng 2013; Paszko et al. 2013; Paszko and Chen in Nobis et al. 2014a; Paszko in Nobis et al. 2014b; Paszko et al. 2017; Paszko and Liu 2018).

In the course of the review of specimens of *Deyeuxia* in three Chinese Herbaria (CDBI, KUN, PE), numerous specimens formerly undetermined or identified as *Deyeuxia pyramidalis* (Host) Veldk. caught the second author’s attention. Most of these specimens were collected in Yunnan, Sichuan and SE Xizang, with a few in adjacent Chinese provinces. For comparison, *Calamagrostis effusiflora* and Eurasian *C. arundinacea* (L.) Roth (in the “Flora of China” as *Deyeuxia pyramidalis* (Host) Veldk.) that show similarity in habit and spikelet traits, were also examined. We concluded that these plants represent an undescribed species new to science and we describe it here as *Calamagrostis hongii* Paszko & Bing Liu.

## Materials and methods

**Figure 1.**
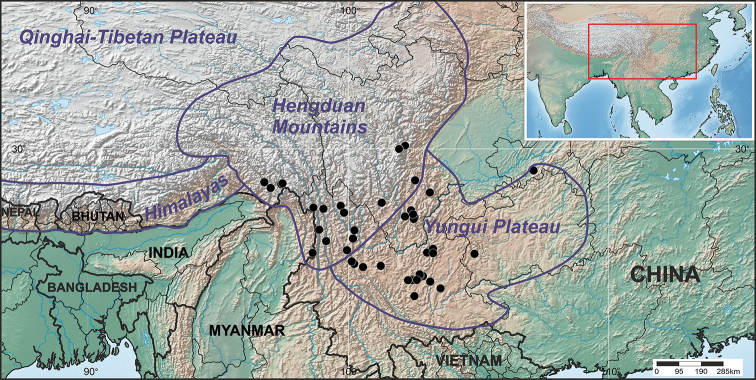
General distribution of *Calamagrostis hongii* Paszko & Bing Liu in southwestern China.

We employed standard techniques for morphological studies of herbarium specimens from the CDBI, KUN, PE, US and W (acronyms follow Thiers 2017). All measurements were taken from the best-developed spikelets and leaf characteristics were determined on the 2^nd^ leaf from the top of the plants. The locality data in accounts below inferred from sources other than herbarium labels are placed in square brackets. The localities were sorted according to the county-level administrative division of the People’s Republic of China. The distribution map was created with SimpleMappr (Shorthouse 2010) (Fig. 1). Specimens with the barcode numbers are accessible online via the PE Herbarium (http://pe.ibcas.ac.cn/en/), the National Plant Specimen Resource Center (http://www.cvh.ac.cn/), or the Muséum National d’Histoire Naturelle (https://science.mnhn.fr/institution/mnhn/collection/p/item/search). The data underpinning the analyses reported in this paper are deposited at GBIF, the Global Biodiversity Information Facility, https://www.gbif.org/dataset/c6dd8791-eaae-49f5-9c18-b4bc06a7357f.

## Taxonomic treatment

### Calamagrostis hongii

Taxon classificationPlantaePoalesPoaceae

Paszko & Bing Liu sp. nov.

6B66FAE0-254E-5097-9F8A-F3D6A13D330F

urn:lsid:ipni.org:names:77212604-1

Figs 1 , 2 , 3

Deyeuxia zhongdianensis L. Liou (Liou 1994: 2235), nom. nud. (Art. 39.1 of the ICN, Turland et al. 2018; no Latin description and/or diagnosis). Cited material: “[CHINA. Yunnan] Zhongdian Co. [now Shangri-La] (K.M. Feng 3326), grassland under forest, riversides, 2700 m”.

#### Diagnosis.

*Calamagrostis hongii* is similar in habit and spikelet morphology to *C. arundinacea* and *C. effusiflora*, but differs in color and hairiness of the upper (adaxial) leaf blade surface. The upper leaf surface of *Calamagrostis hongii* is characterized by the grey color (vs. green or grey-green in *C. arundinacea* and *C. effusiflora*), the presence of moderately impressed veins forming ribs (vs. leaf surface flat or veins only slightly impressed in *C. arundinacea* or veins moderately or distinctly impressed, forming ribs in *C. effusiflora*), the presence of numerous prickle hairs covering the ribs (vs. lack of prickle hairs on veins or ribs of *C. arundinacea* and *C. effusiflora*) and the absence of hairs (vs.moderately or densely hairy in *C. effusiflora* and slightly or not hairy in *C. arundinacea*). *Calamagrostis hongii* is characterized by glabrous leaf sheaths, blades and collars.

#### Type.

China • **Yunnan**: Shilin Co., Guishan, Haiyi village to Yumeidu village; alt. 2095 m; 24.647N, 103.542E; 18 August 2006; Y.M. Shui et al. 64471 (Holotype PE! [herb. no. 2308966], Isotypes PE! [herb. no. 2058824, 2070270].

#### Description.

***Perennial grass***, cespitose, without rhizomes. ***Culms*** 55–140 cm tall, erect, unbranched above, 3–4.5 mm in diameter near the base, nodes 3–5, glabrous below the panicle. ***Leaf sheaths*** glabrous; ***collar*** glabrous; ***ligules*** 1.9–10 mm long, acute; ***blades*** 5–55 cm long, 4.3–9.5 mm wide, flat, slightly ribbed with glabrous furrows and scabrous ribs, upper (adaxial) surface scabrous owing to the presence of short stiff prickles on ribs, gray or gray–green, lower surface slightly scabrous, green, scabrous along margins. ***Panicles*** 13–25 cm long, erect, open, or loosely contracted at maturity; proximal internode 0.6–3.7(–4.5) cm long; rachis with 3–7 branches per node; ***branches*** 4–10 cm, slightly scabrous, spikelet-bearing only beyond mid-length. ***Spikelets*** 3.8–6.6 mm long, 1-flowered, with one fertile floret with rachilla extension, laterally compressed, disarticulation above the glumes; ***glumes*** subequal or equal, glabrous, very weakly scabrid on keel, apex acuminate; ***lower glumes*** 3.8–6.6 mm long and 0.9–1.4 mm wide, 1-veined; ***upper glumes*** 3.5–5.9 mm long and 1.1–1.6 mm wide, 3-veined, 0.8–1.1 times as long as the lower glume; ***callus hairs*** 1.0–2.9 mm long, 0.3–0.6 times as long as the lemmas; ***lemmas*** 3.5–4.8 mm long, 5-veined, 0.7–1.0 times as long as the lower glumes, apex 4-toothed; ***lemmatal awn*** 5.3–7.8 mm long, arising from near base (0.06–0.15 way up the back) of the lemmas, exserted, slender and easily distinguished from the callus hairs, geniculate, with twisted column; ***paleas*** 2.8–4.5 mm long, subequal or equal to the lemma, 0.8–1.0 times as long as the lemmas; ***rachilla*** extensions 1.0–2.8 mm long, densely bearded with hairs 2.4–4.2 mm; ***stamens*** 3, ***anthers*** 1.3–2.6 mm long. ***Fl.*** Jul–Aug. ***Fr.*** Aug–Sep.

**Figure 2.**
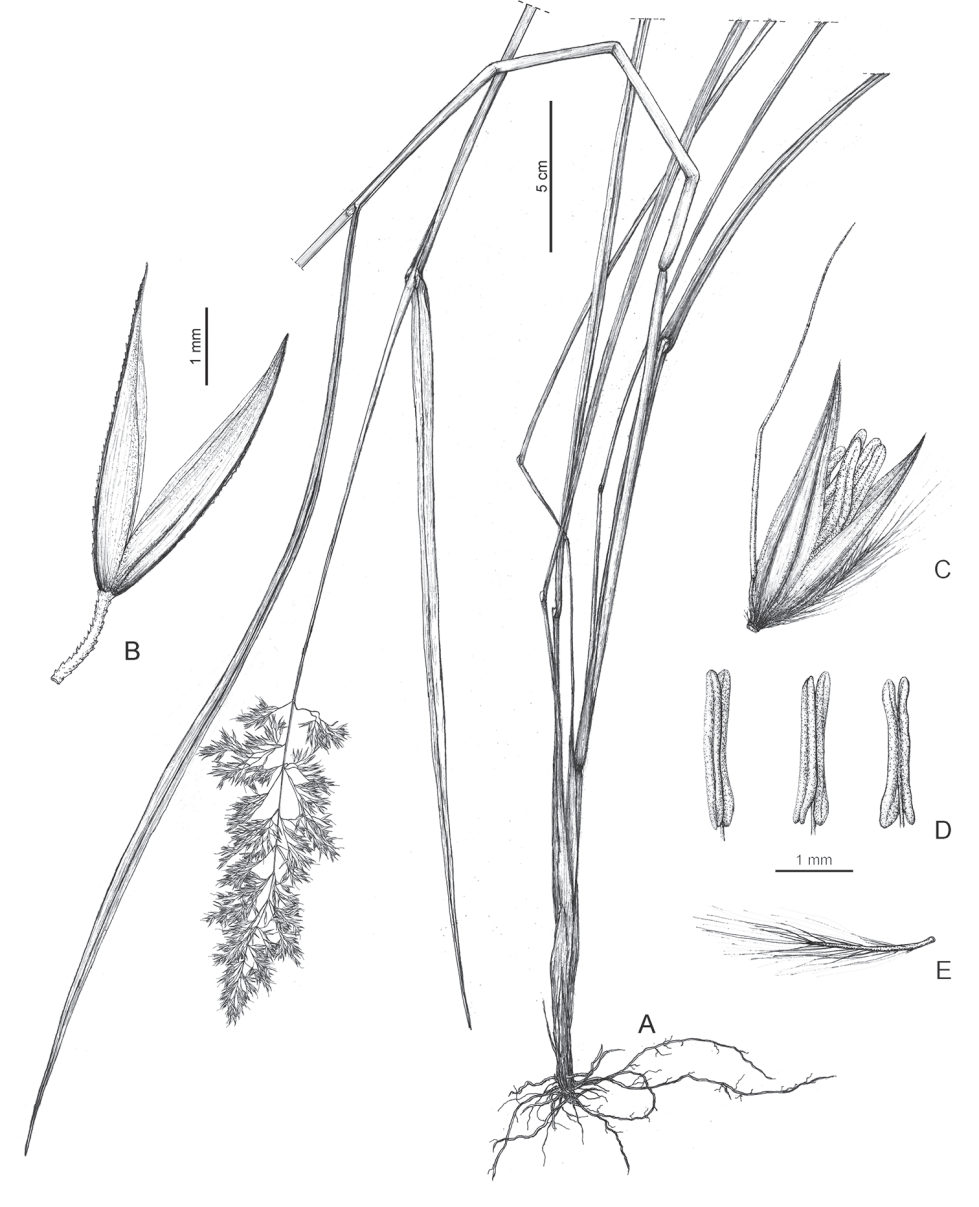
Illustration (drawn by Jolanta Urbanik) of *Calamagrostis hongii* Paszko & Bing Liu based on Y.M. Shui et al. 64471 (PE, herb. no. 2308966) from Shilin County (Yunnan, China) **A** habit **B** glumes **C** floret **D** anthers **E** rachilla prolongation. Scale bars: 5 cm (**A**); 1 mm (**B–E**).

#### Taxonomic note.

**Figure 3.**
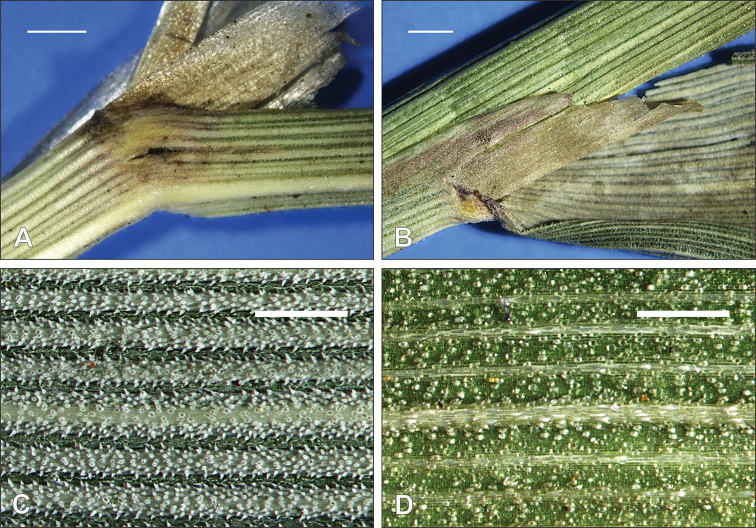
Leaf (2^nd^ leaf from the top) characteristics of *Calamagrostis hongii* Paszko & Bing Liu **A** glabrous leaf collar **B** leaf ligule **C** upper (adaxial) leaf surface **D** lower (abaxial) leaf surface. **A–D** China, Yunnan: Shilin Co., Y.M. Shui et al. 64471 (PE, herb. no. 2308966). Scale bars: 1 mm (**A, B**); 0.5 mm (**C, D**). Photographs by B. Paszko.

**Figure 4.**
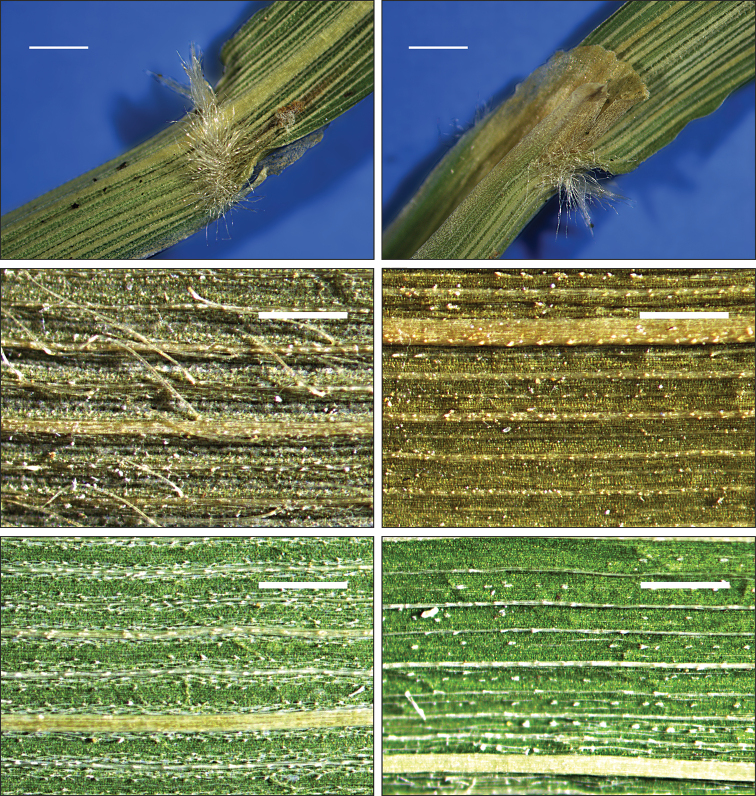
Leaf (2^nd^ leaf from the top) characteristics of *Calamagrostis arundinacea* (L.) Roth. **A** hairy leaf collar **B** leaf ligule **C** upper (adaxial) surface **D** lower (abaxial) surface. **A–D** Slovakia, V. Mikoláš 8501 (W) **E, F** France, G. Gautier 63 (W). Scale bars: 1 mm (**A, B**); 0.5 mm (**C, D**). Photographs by B. Paszko.

**Figure 5.**
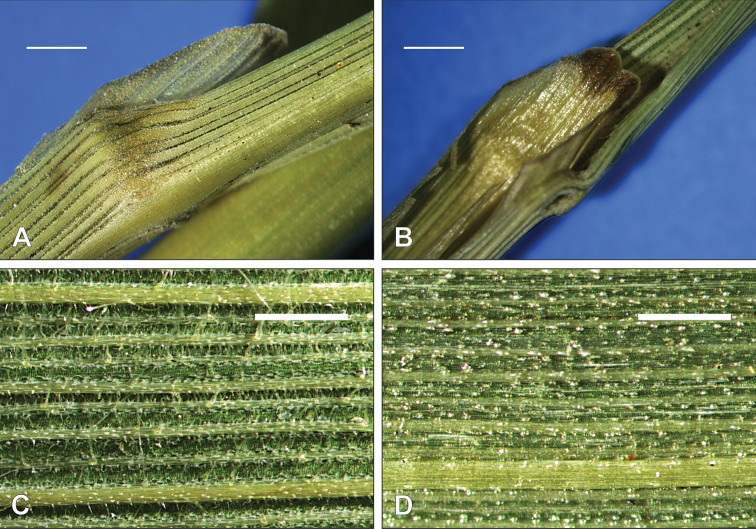
Leaf (2^nd^ leaf from the top) characteristics of *Calamagrostis effusiflora* (Rendle) J.L.Yang **A** leaf collar **B** leaf ligule **C** upper (adaxial) surface **D** lower (abaxial) surface. **A–D** China, Sichuan, Wenchuan Co., Wolong Nature Reserve, 1500 m, 1 September 1982, K.Y. Lang et al. 1424 (PE01727049). Scale bars: 1 mm (**A, B**); 0.5 mm (**C, D**). Photographs by B. Paszko.

Although *Calamagrostis* and *Deyeuxia* were revised for China only fourteen years ago in the “Flora of China” (Lu and Phillips 2006, Lu et al. 2006) it is necessary to present a new taxonomic account of these species because of the changes in generic circumscription and the description of new species. *Calamagrostis hongii* is the third new species of *Calamagrostis* reported from China since the publication of the “Flora of China”. This new species is probably a member of the *C. arundinacea* complex and it is similar to *C. arundinacea* and *C. effusiflora* in habit and spikelet traits, including size of glumes, lemmas and paleas, presence of well-developed rachilla prolongation and a geniculate lemma awn with a twisted basal column, lemma awn length and its insertion on the lemma back (near the base to the lower 1/3). However, they differ in several diagnostic characteristics. Prior to this study, most of the specimens currently identified as *C. hongii* had been identified as *Deyeuxia pyramidalis* [= *Calamagrostis arundinacea*]. The detailed revision of this group of species by the present authors showed that the upper surfaces of the leaves of *C. hongii* are unique. They are gray in color and moderately or densely scabrous and characterized by the presence of moderately impressed veins forming ribs that are slightly or densely covered by numerous prickle hairs (Fig. 3). Such prickle hairs are absent from the upper surfaces of the leaf blades of *C. arundinacea* and *C. effusiflora*. The upper surface of the leaf blade of *C. arundinacea* has veins that are only slightly impressed and the leaf blade surface is almost flat and hairless or covered by scattered macro hairs (Fig. 4), whereas *C. effusiflora* has veins slightly to distinctly impressed, forming ribs that are usually moderately to very densely hairy (Fig. 5). All three species have prickly leaf edges. The prickle hairs have thick walls that can be silicified. For additional diagnostic characteristics see Table 1.

**Table 1.** Diagnostic morphological characters of *Calamagrostis hongii*, *C. arundinacea*, and *C. effusiflora*.

**character**	***C. hongii***	***C. arundinacea***	***C. effusiflora***
Panicle length (cm)	12–25	12–28	13–43
Rachilla length (mm)	1.0–2.75	0.75–1.75	0.25–2.0
Rachilla length with hairs (mm)	2.4–4.4	1.55–4.25	1.5–4.0
Anther length (mm)	1.35–2.6	2.15–3.3	1.2–2.75
Leaf ligule length (mm) at the 2^nd^ leaf from the top	1.9–10.3	0.9–5.5	0.8–18.0
Ratio: palea to lemma length	0.8–1.0	0.7–1.1	0.8–1.0
Colour of upper (adaxial) leaf blade surface	grey	green	grey-green, rarely green
Leaf veins on the upper (adaxial) leaf blade surface (in the middle of a leaf)	moderately impressed, forming ribs	only slightly impressed, leaf surface flat or almost flat	slightly to distinctly impressed, forming ribs
Upper (adaxial) leaf blade surface	moderately to densely scabrous, glabrous	smooth or covered with scattered macro hairs	slightly to densely hairy, macro hairs absent or present
Lower (abaxial) leaf blade surface	slightly scabrous	slightly scabrous	slightly scabrous
Presence of prickle hairs on leaf edges	present	present	present
Leaf collar (the junction of the leaf sheath and blade)	always glabrous	usually hairy, very rarely glabrous	usually glabrous, rarely hairy, the collar may also be found with a hairy margin
Altitude (m)	1800–3350	0–2300	600–2900(?)

The designation *Deyeuxia zhongdianensis* L. Liou (Liou 1994: 2235) was described without Latin description or diagnosis. From January 1, 1935, to December 31, 2011, one or both had to be in Latin, thus *Deyeuxia zhongdianensis* is nomenclaturally invalid and therefore unavailable under the Article 39.1 of the ICN (Turland et al. 2018). Liou (1994: 2235) cited Feng’s collection no. 3326 from Zhongdian County (now Shangri-La) in Yunnan Province, but the herbarium was not specified by the author. In PE we located three herbarium sheets (PE01854125, PE02108399, PE02108400) collected at Mt. Wuzhujun at Shangri-La City (former Zhongdian Co.) in Yunnan Province. We identified Feng’s collection as *C. hongii*. Lu et al. (2006) incorrectly synonymized *Calamagrostis zhongdianensis* with *Deyeuxia pyramidalis* (= *C. arundinacea*).

#### Vernacular name.

洪氏野青茅(Chinese), Hong’s Bent-grass (English).

#### Etymology.

The specific epithet honors Professor De-Yuan Hong, the Academician of Chinese Academy of Sciences (CAS) (State Key Laboratory of Systematic and Evolutionary Botany, Institute of Botany, CAS, Beijing, China) for his outstanding achievements in systematics, morphology, cytology, ecology and molecular evolution. The second author thanks Professor De-Yuan Hong for his continuous support during her multiple research visits to the Chinese herbaria as part of an exchange program between the Polish Academy of Sciences and the Chinese Academy of Sciences, in order to study the herbarium collections of *Calamagrostis* and *Deyeuxia* for the “Flora of Pan-Himalaya”.

#### General distribution.

China (S Chongqing, W Guizhou, Sichuan, SE Xizang, Yunnan).

#### Distribution and habitat.

*Calamagrostis hongii* is endemic to south-western China. It is centered on the northern part of Yunnan and southern part of Sichuan and adjacent regions in south-western and central China, such as southern Chongqing, western Guizhou, central Sichuan and south-eastern Xizang. Its distribution covers the Southern Hengduan Mts and western and northern part of Yungui Plateau. A dot map provided here (Fig. 1) shows it to be common (or at least commonly collected) in northwestern Yunnan, with three dots along the Chinese border with Kachin State of Myanmar, where it may also occur. *Calamagrostis hongii* is restricted to the Sino-Himalayan subkingdom, primarily the Yunnan Plateau and Hengduan Mountains (Peng and Wu 2013, Tang 2015). The species occurs in the montane belt from circa 1800 m to 3350 m a.s.l., in grasslands, among bushes, forest edges and in mixed *Pinus yunnanensis* forests.

#### Phenology.

*Calamagrostis hongii* flowers from July to August and is in fruit from August to November.

#### Additional specimens examined.

China – **Chongqing** • **Nanchuan** Distr.; [29.168N, 107.105E]; 31 Oct. 1960; Nanshuibeidiao Exped. Team 4852 leg.; KUN (KUN0079536). – **Guizhou** • [**Panzhou** City (Panxian Co., Pan Co.)]; Mt. Bada; mountain top, bushy and sunny place; alt. 2620 m; [25.975N, 104.839E]; 22 Aug. 1959; Anshun Exped. Team 1119 leg.; KUN (KUN0081068), PE. – **Sichuan** • **Dechang** Co.; [27.405N, 102.173E]; 1 Sept. 1959; S.F. Zhu 20177 leg.; PE (PE01726895); • **Kangding** City, Guzan; alt. 2200 m; [30.12N, 102.177E]; 6 Aug. 1961; Nanshuibeidiao Exped. Team 9903 leg.; PE (PE01726896) • **Kangding** City, [Xinduqiao Township], (Thibet Orient.) Tongolo [东俄洛, Dong’eluo] (Principauté de Kiala); [30.079N, 101.48E]; Jun.–Jul. 1892; R.P. Soulié s.n. leg.; P (P02650457, P02650453), PE (PE00449806) • **Meigu** Co.; mixed forest; alt. 2220 m; [28.325N, 103.127E]; 3 Aug. 1959; Z.T. Guan 7448 leg.; PE (PE00449784) • **Muli** Co., Zhongmi; alt. 2700 m; [27.925N, 101.263E]; 18 Jul. 1978; K.H. Mou, Y.B. Yang 7390 leg.; CDBI (CDBI0154069) • **Muli** Co., alt. 2800–3350 m, [27.934N, 101.28E], 15 Sept. 1959, S.K. Wu 3261 leg.; KUN (KUN0081354), PE (PE00449779) • **Puge** Co., Tuomugou; forest, slope; alt. 1800 m; [27.381N, 102.54E]; 28 Aug. 1959; s.c. 5589 leg.; KUN (KUN0079385), PE (PE00449783) • **Puge** Co., Li’an; sunny slope; [27.312N, 102.508E]; 18 Aug. 1959; s.c. 5427 leg.; KUN (KUN0079386), PE (PE01726900) • **Puge** Co., Qiaowo Farm; alt. 1600 m; [27.489N, 102.483E]; 7 Aug. 1976; s.c. 14197 leg.; CDBI (CDBI0154083); PE (PE01727010) • **Yuexi** Co., Bao’an; alt. 2000 m; [28.79N, 102.561E]; 12 Jul. 1959; s.c. 3863 leg.; CDBI (CDBI0154071), PE (PE01726894) • unknown locality; alt. 2700 m; 30 Oct. 1965; Xizang Exped. Team s.n. leg.; PE (PE01727001). – **Xizang**: • **Zayü** Co. [formerly known as Tsarung], Tsawarung, Nar-jou; *Pinus yunnanensis* forest; alt. 3300 m; [28.675N, 97.476E]; Sept. 1935; C.W. Wang 66441 leg.; KUN (KUN0081350) • **Zayü** Co., Xiachayu; alt. 2400–2600 m; [28.499N, 97.02E]; 30 Aug. 1983; B.S. Li et al. 7165 leg.; PE (PE01726905) • **Zayü** Co., Zhuwagen; alt. 2500 m; [28.64N, 97.425E]; 8 Aug. 1973; s.c. 652 leg.; PE • **Zayü** Co., Shangchayu; alt. 2000 m; [28.718N, 96.777E]; 20 Aug. 1979; T.P. Yi 79153 leg.; KUN • **Zayü** Co., Shangchayu; alt. 2200 m; [28.718N, 96.777E]; 24 Jul. 1980; Z.C. Ni et al. 0724 leg.; PE (PE00449813, PE00449814) • **Zayü** Co.; Hougou; alt. 2300–2600 m; 26 Aug. 1983; B.S. Li et al. 6827 leg.; PE (PE01727016)). – **Yunnan**: • **Anning** City, Mt. Bijia, bushes, limestone, alt. 2200 m, [24.993N, 102.459E]; 2 Sept. 1977; B.Y. Qiu 77844 leg.; CDBI (CDBI0154080), KUN (KUN0097423) • **Anning** City, Wenquan Town; pine forest; alt. 1880 m; [24.962N, 102.45E]; 14 Aug. 2006; E.D. Liu 1807 leg.; KUN (KUN0397297) • **Anning** City, Anfengying; [24.966N, 102.289E]; 22 Jul. 2007; Y.C. Liu, J. Xu 119 leg.; KUN (KUN1221260) • **Dali** City, Ta-li Hsien; pine forest; alt. 2400 m; [25.589N, 100.226E]; 28 Jul. 1933; H.T. Tsai 53898 leg.; KUN (KUN0081049), PE(PE00449810) • **Dali** City, Mt. Cangshan; grassland; [25.589N, 100.226E]; 3 Aug. 1963; Zhongdian Exped. Team 63-3845 leg.; KUN (KUN0081048) • **Dali** City, Mt. Cangshan; alt. 3000 m; [25.589N, 100.226E]; 1 Oct. 2002; H.Y. Ma 153 leg.; KUN (KUN0081035) • **Dali** City, Mt. Cangshan; [25.467N, 100.56E]; 20 Jul. 1906; F. Ducloux 4223 leg.; PE • **Dali** City, Mt. Cangshan, Zhonghe Temple; [25.68N, 100.132E]; 30 Nov. 1948; T.N. Liou 17405 leg.; PE (PE00449804) • **Dali** City; without precise locality; Sept. 1941; H.C. Wang 1386 leg.; PE (PE00449803) • **Dongchuan** Distr., Kunming, Fazhecun, Damufang; [26.023N, 103.021E]; 16 Aug. 1964; Diandongbei Exped. Team 811 leg.; KUN (KUN0081296) • **Eryuan** Co.; alt. 2600 m; [26.113N, 99.949E]; 31 Sept. 1963; W Yunnan-Jinsha River Exped. Team 63-6292 leg.; KUN • **Eryuan** Co., without precise locality; alt. 2600 m; [26.113N, 99.949E]; W Yunnan-Jinsha River Exp. Team 63-6292 leg.; KUN (KUN0081353), PE (PE01726887, PE01726893) • **Fugong** Co., Famufang, forest; alt. 2700 m; [26.901N, 98.88E]; 23 Jun. 1978; Bijiang Exped. Team 715 leg.; KUN (KUN0097433) • **Gongshan** Co., Suroula; alt. 3000 m; [27.748N, 98.662E]; Sept. 1935; C.W. Wang 66586 leg.; PE (PE00449807) • **Guandu** Distr., Kunming, Shuanglong; grassland; alt. 1900 m; [25.119N, 102.862E]; 1 Sept. 1977; B.Y. Qiu 77700 leg.; CDBI (CDBI0154079), KUN (KUN0079418, KUN0079425) • **Heqing** Co., Huangping, Junle, Shangdapingzi; alt. 2500 m; [26.559N, 100.179E]; 16 Aug. 1963; Jinshajiang Exped. Team 6555 leg.; KUN (KUN0081171), PE (PE01726857, PE01726891) • **Heqing** Co., Les paturages au col de Koua-la-po, pres Hokin; alt. 3000 m; 1883–1885; M. Delavay 2465 leg.; P (P02650445), PE (PE01938085, PE01663486), W (1916-38022) • **Huize** Co., Liangwang Shan, ca. 15 km E of Dongchuan ca. 120 km NNE of Kunming, on new rd. to Zhoatong via Zhehai; shallow grassy valley in low, red clay hills, with limestone substrate surrounded by 2^nd^ growth *Pinus yunnanensis*, *Alnus nepalensis*, *Quercus variabilis* and *Q. glaucoides* forest, *Capillipedium* abundant; alt. 2280 m; 26.167N, 103.25E; 14 Sept. 1997; R.J. Soreng et al. 5309 leg.; KUN (KUN0079581), PE (PE00487541), US (US00895217) • **Huize** Co., Liangwang Shan, ca. 20 km E of Dongchuan, ca. 110 km NNE of Kunming, on new rd. to Zhoatong via Zhehai; low, erroded, red clay hills, with limestone substrat, scrubby *Pinus yunnanensis*/*Alnus nepalensis* forest, shrubs and grasses, around corn and tobacco fields, among trees; 26N, 103.25E; 14 Sept. 1997; R.J. Soreng et al. 5293 leg.; KUN (KUN0079580), US (US00844386) • **Jiangchuan** Distr., Yuxi City, Cuifeng, Yongsheng; limestone; [24.355N, 102.533E]; 9 Aug. 1975; B.Y. Qiu 60633 leg.; KUN (KUN0081051) • **Lanping Co.**, Yingpan, Luomuping; alt. 2300 m; [26.464N, 99.149E]; 21 Jun. 1981; PE Mt. Hengduan Exped. Team 683 leg.; PE (PE01663487) • **Lushui** City, Pianma Town; grassland of forest edge; alt. 2100 m; [26.012N, 98.63E]; 30 Jul. 1978; Bijiang Exped. Team 1542 leg.; KUN (KUN0081066) • **Panlong** Distr., Kunming, Heilongtan; [25.141N, 102.751E]; 1945; K.M. Feng 10636 leg.; KUN (KUN0081054) • **Panlong** Distr., Kunming, Heilongtan; shady slope among high herbs; [25.141N, 102.751E]; 26 Jul. 1941; F.T. Wang 2286 leg.; KUN (KUN0081089) • **Weishan** Co., Huangshucun; alt. 2370 m; 22 May 1965; CAS Integrated Exped. Team for SW China 449 leg.; PE • **Weixi** Co., Wei-si Hsien, Yeh-Chih [Yezhizhiang]; ravine; alt. 2400 m; [27.699N, 99.044E]; Jul. 1935; C.W. Wang 67940 leg.; KUN (KUN0081351) • **Wuhua** Distr., Kunming, Xiaoshao; 25.186N, 102.735E; 18 Jul. 2007; H. Peng et al. 68 leg.; KUN (KUN1221262) • Zhongdian Co. [now **Shangri-La** City], Mt. Wuzhujun; forest; alt. 2700 m; [27.817N, 99.707E]; 12 November 1939; Feng 3326 leg.; PE (PE02108400, PE02108399, PE01854125) • Zhongdian Co. [now **Shangri-La** City], Xiaozhongdian, East Mt.; alt. 3250 m; [27.55N, 99.835E]; 13 Aug. 1981; s.c. W16 leg.; PE (PE01726860) • Zhongdian Co. [now **Shangri-La** City]; alt. 2900 m; 9 Jul. 1962; Zhongdian Exped. Team 2106 leg.; PE (PE01726889) • **Xishan** Distr., Kunming, Hsi-Shan [Xishan], near San-Ching-Ke; grassland; alt. 2100 m; [24.97N, 102.627E]; 11 Aug. 1945; T.N. Liou 14085 leg.; PE (PE00449812) • **Xishan** Distr., Kunming, Western Hills; [24.974N, 102.628E]; 1941; T.N. Liou 19823 leg.; PE (PE00449802) • **Yao’an** Co., Daxingshan; alt. 2180 m; [25.514N, 101.242E]; 11 Jul. 1965; CAS Southwest Exped. Team 484 leg.; PE • **Yi-liang** Co., way to Yangzonghai; alt. 1800 m; [24.911N, 103.142E]; 6 Sept. 1977; B.Y. Qiu 771257 leg.; CDBI (CDBI0154088) • **Yulong** Co., Yangtze Watershed, Prefectural District of Lijiang, eastern slopes of Lijiang Snow Range; [26.877N, 100.234E]; May–Oct. 1922; J.F. Rock 5911 leg.; PE • same collection data as for preceding; J.F. Rock 5908 leg.; P (P02650461), W • **Yulong** Co., Lijiang (Likiang), Mt. Yulong-schan; Jun–Sept. 1914–1916; Handel-Mazzetti s.n. leg., Inter Sinense 1914–1918 no. 3790; W • **Yongsheng** Co., Boluo; edge of *Pinus yunnanensis* forest; alt. 2600 m; 16 Jul. 1960; Sino-Russia Exped. Team 6205 leg.; PE (PE01726892).

## Supplementary Material

XML Treatment for Calamagrostis hongii
